# The independent association between parathyroid hormone levels and hyperuricemia: a national population study

**DOI:** 10.1186/ar3769

**Published:** 2012-03-10

**Authors:** Janet Y Hui, Jee Woong J Choi, David B Mount, Yanyan Zhu, Yuqing Zhang, Hyon K Choi

**Affiliations:** 1 The Clinical Epidemiology Unit, Boston University School of Medicine, 650 Albany Street, Suite 200, Boston, MA 02118, USA; 2 Renal Division, Department of Medicine, Brigham and Women's Hospital, Harvard Medical School, 4 Blackfan Circle, Boston, MA 02115 and Nephrology Division, VA Boston Healthcare System, 150 S. Huntington Ave, Boston, MA 02130, USA; 3 Section of Rheumatology, Boston University School of Medicine, 650 Albany Street, Suite 200, Boston, MA 02118, USA

## Abstract

**Introduction:**

Increased frequencies of hyperuricemia and gout have been associated with primary hyperparathyroidism, and recent clinical trials of parathyroid hormone (PTH) have reported hyperuricemic adverse events. We evaluated the potential population impact of PTH on serum uric acid (SUA) levels by using a nationally representative sample of United States adults.

**Methods:**

By using data from 8,316 participants aged 18 years and older in the National Health and Nutrition Examination Survey 2003 to 2006, we examined the relation between serum PTH and SUA levels with weighted linear regression. Additionally, we examined the relation with hyperuricemia by using weighted logistic regression.

**Results:**

SUA levels increased with increasing serum PTH concentration. After adjusting for age, sex, dietary factors, glomerular filtration rate (GFR), and other potentially related biomarkers (calcium, phosphorus, alkaline-phosphatase, 25-hydroxyvitamin D), the SUA level differences from the bottom (referent) to top quintiles of serum PTH levels were 0, 8, 13, 14, and 19 μ*M *(95% CI, 12 to 26; *P *for trend, < 0.001). These estimates were larger among renally impaired individuals (multivariate SUA difference between the extreme quintiles of PTH, 26 versus 15 μ*M *among those with GFR ≥ 60 versus < 60 ml/min per 1.73 m^2^, respectively) (*P *for interaction = 0.004). The odds of hyperuricemia by various definitions increased with increasing PTH levels as well (multivariate *P *values for trend, < 0.05).

**Conclusions:**

These nationally representative data indicate that serum PTH levels are independently associated with serum uric acid levels and the frequency of hyperuricemia at the population level.

## Introduction

Hyperuricemia is the precursor of gout, a common and painful inflammatory arthritis with a growing disease burden [1]. Hyperuricemia and gout have been long observed to occur with an increased frequency among patients with hyperparathyroidism, whereas parathyroidectomy is known to reduce serum uric acid levels in these cases [2-7]. Elevated parathyroid hormone (PTH) levels are thought to reduce renal urate excretion, although the exact mechanism remains unclear [7]. Furthermore, recent randomized controlled trials have also found that hyperuricemia or gout is associated with use of teriparatride, a recombinant human PTH approved for osteoporotic fracture prevention [8-10]. After cessation of teriparatride, serum uric acid levels decrease [8]. The baseline data of a randomized trial based on men aged > 40 years [11] and another community population study of men aged ≥ 70 years [12] showed a significant association between PTH and serum uric acid levels. These data suggest that a significant biologic influence of PTH on serum uric acid level exists, but the impact of this relation at the general population level (including women) is not known. To address this issue, we examined the relation between serum PTH and uric acid levels in a nationally representative sample of men and women (US National Health and Nutritional Examination Survey, NHANES 2003 to 2006).

## Methods

### Study population

The NHANES studies are designed to assess the health and nutritional status of adults and children in the United States. These studies are based on a representative sample of the noninstitutionalized US civilian population that is selected by using a multistage, stratified sampling design. The survey is unique in that it combines interviews, physical examinations, and various laboratory data [13]. Beginning in 1999, NHANES became a continuous survey conducted annually with data released in 2-year cycles. In this analysis, we used two recent NHANES datasets that obtained serum PTH levels, the NHANES 2003-2006. After a home interview, participants were invited to attend examination sessions, in which blood and urine specimens were obtained. Our analysis included participants 18 years or older who attended the medical examination, and included the 8,316 participants (3,961 men, 4,355 women) with complete information. We repeated our analysis among 8,272 participants, after excluding participants who were taking allopurinol or uricosuric agents (*n *= 44).

The NHANES studies underwent institutional review board approval, and written informed consent was obtained from participants before starting the study.

### Measurements

PTH levels were measured by using the Origen-Electrochemiluminescent analysis (Elecsys 1010 Immunoassay analyzer). The analytic protocols for other serum analytes and laboratory quality-assurance procedures are described in the NHANES Laboratory Procedures Manuals [14]. Values are reported in picomoles per liter, and can be converted to picograms per microliter by dividing by 0.1061.

Serum uric acid levels were measured by oxidization with the specific enzyme uricase to form allantoin and H_2_O_2 _(Beckman Synchron LX20). Details about quality-control procedures were been published in the NHANES Laboratory Procedures Manuals [14]. Values are reported in micromoles per liter and can be converted to milligrams per deciliter by dividing by 59.48.

### Assessment of covariates

The NHANES collected information on body measurements (including body mass index (BMI)), medication use (including diuretics, allopurinol, and uricosuric agents), medical conditions (including self-reported hypertension), and relevant laboratory values (serum creatinine, calcium, alkaline phosphatase, phosphorus, and 25-hydroxyvitamin D levels). Glomerular filtration rate (GFR) was estimated by using the Modification of Diet in Renal Disease study equation: $\text{GFR}\;(\text{ml}/\text{min}\;\text{per}\;\text{1}.\text{73}\;{\text{min}}^{\text{2}})=\text{186}\times {\left(\text{serum}\;\text{creatinine}\;\text{level}\;\text{(mg}/\text{dl)}\right)}^{-\text{1}.\text{154}}\times {\left(\text{age}\right)}^{-.\text{2}0\text{3}})\times (0.0\text{742},\;\text{if}\;\text{female})\times (\text{1}.\text{212},\;\text{if}\;\text{ black})$[15-17].

The average daily intakes of energy, protein, carbohydrates, sugar, fat, caffeine, and alcohol were derived from a mean of two 24-hour dietary recalls conducted two separate days after the medical examination.

### Statistical analysis

All statistical analyses were computed by using survey commands of STATA (for example, SVYMEAN and SVYREG) to incorporate sample weights and to adjust for clusters and strata of the complex sample design (STATA Corporation, College Station, TX, USA).

We used linear regression modeling to evaluate the relation between PTH levels and serum uric acid levels. PTH levels were categorized into quintiles, and each quintile was compared with the lowest quintile. Multivariate models were adjusted for: age, sex, race, BMI, self-reported hypertension, use of diuretics, use of allopurinol or uricosuric agents, GFR, intake of alcohol, total energy, protein, and sugar, and mutually for serum levels of PTH, calcium, alkaline phosphatase, phosphorus, and vitamin D. To help address the potential influence of central adiposity (reflecting insulin resistance), we conducted analyses with adjustment for waist circumference in addition to, as well as in place of, BMI. Trends in serum uric acid levels across PTH categories were assessed in linear regression models by using continuous PTH values. We performed logistic regression with a dichotomous outcome of hyperuricemia (serum uric acid level > 7.0 mg/dl in men and > 5.7 mg/dl in women [18]), adjusting for the same covariates. We also examined the potential impact of alternative definitions of hyperuricemia (serum uric acid level > 6.0 mg/dl and > 7.0 mg/dl, both regardless of sex) in these regression models.

We examined whether the observed associations persisted within the subgroups stratified by sex, BMI (< 25 kg/m^2 ^versus ≥ 25 kg/m^2^), GFR (≥ 60 versus < 60 ml/min per 1.73 m^2^), serum vitamin D levels (≥ 52.4 n*M *(median value) versus < 52.4 n*M*), and serum calcium levels (≥ 2.4 m*M *(median value) versus < 2.4 m*M*). We determined the statistical significance of potential subgroup effects by testing the significance of interaction terms added to our final multivariate models. For all difference estimates and odd ratios, we calculated 95% confidence intervals (CIs). All *P *values are two-sided.

## Results

The mean age of the population was 46 years. The mean serum uric acid level was 319.05 μ*M *(361 μ*M *among men and 280 μ*M *among women), and 19.13% were hyperuricemic (20.86% among men and 17.54% among women). The characteristics of the study according to PTH level are shown in Table 1. With increasing PTH levels, age, BMI, serum levels of alkaline phosphatase, use of diuretics and urate-lowering agents, and the frequency of hypertension and diabetes tended to increase, whereas GFR, the proportion of whites, and serum levels of calcium, vitamin D, and phosphorus tended to decrease. Furthermore, intake of total energy, carbohydrates, protein, sugar, fat, and caffeine tended to decrease with increasing PTH levels.

**Table 1:** Characteristics according to quintiles of parathyroid hormone (PTH) levels in NHANES 2003-2006^a^

**Quintiles of serum PTH levels**, p*M* (pg/ml)	*n*	Age (years)	Men (%)	White (%)	**BMI (kg/m**^**2**^)	Diuretic use (%)	Serum calcium (m*M*)	Serum vitamin D (n*M*)	Serum phosphorus (m*M*)	Serum alkaline phosphatase (U/L)	**Uric acid drug use**^**b**^ (%)	History of hypertension (%)	GFR (ml/min per 1.73 m^2^)	Alcohol (g/day)	Total energy (kcal/day)	Total protein (g/day)	Total sugar (g/day)	Total caffeine (mg/day)
0.6-2.9 (6-27)	1,733	40	47	80	26.8	4.2	2.4	69.2	1.3	65.9	0.1	23	96	10.9	2263	87.9	129.3	185.1
3.0-3.8 (28-36)	1,795	44	50	76	27.5	4.9	2.4	63.0	1.3	66.8	0.2	28	93	9.9	2179	84.3	120.8	179.3
3.9-4.7 (37-44)	1,462	47	49	75	28.3	5.7	2.4	59.4	1.3	67.0	0.1	29	91	9.9	2192	84.8	119.3	186.1
4.8-6.2 (45 - 59)	1,712	49	47	70	29.1	7.5	2.4	55.2	1.2	70.0	1.0	41	89	8.5	2129	83.8	117.0	157.2
6.3-52.1 (59-491)	1,614	51	46	66	30.4	14.7	2.4	49.2	1.2	74.2	1.1	48	83	6.3	2009	77.2	115.0	156.7
All	8,316	46	48	74	28.3	7.0	2.4	59.8	1.2	68.5	0.5	33	91	9.2	2160	83.9	120.6	173.5

^a^Data are presented incorporating sample weights and adjusted for clusters and strata of the complex sample design of NHANES 2003-2006. ^b^Allopurinol and uricosuric agents.

After adjusting for age and sex, serum uric acid levels among individuals in the highest quintile were higher than those in the lowest quintile by 36 μ*M *(95% CI, 28 to 44; *P *for trend, < 0.001). After adjusting for other covariates, the difference was attenuated to 19 μ*M *(95% CI, 12 to 26; *P *for trend < 0.001), but remained significant (Table 2). When we repeated our analyses after excluding participants who were taking allopurinol or uricosuric agents (*n *= 44), the results did not significantly change. When we repeated our multivariate analyses with adjustment for waist circumference, additionally or in place of BMI, our results did not change materially (*P *values for trend < 0.001).

**Table 2:** Differences in serum uric acid levels according to quintiles of parathyroid hormone in NHANES 2003-2006

Parathyroid hormone levels p*M* (pg/ml)	Quintile 1 0.64-2.86 (6-27)	Quintile 2 2.97-3.82 (28-36)	Quintile 3 3.93-4.67 (37-44)	Quintile 4 4.77-6.15 (45-58)	Quintile 5 6.26-52.10 (59-491)	P value for trend
Age- and sex-adjusted difference, μ*M* mg/dl	0 (referent)	8.9 (3.3, 14.6) 0.15 (0.05, 0.25)	15.2 (9.1, 21.3) 0.26 (0.15, 0.36)	20.3 (14.0, 26.6) 0.34 (0.24, 0.45)	35.9 (28.0, 43.8) 0.60 (0.47, 0.74)	< 0.001
Multivariate difference,^a ^μ*M* mg/dl	0 (referent)	7.6 (3.0, 12.2) 0.13 (0.05, 0.21)	12.6 (6.7, 18.5) 0.21 (0.11, 0.31)	14.2 (8.6, 19.8) 0.24 (0.14, 0.33)	22.9 (16.3, 29.4) 0.39 (0.27, 0.49)	< 0.001
Multivariate difference,^b ^μ*M* mg/dl	0 (referent)	7.3 (2.9, 11.7) 0.12 (0.05, 0.20)	13.2 (7.4, 19.0) 0.22 (0.12, 0.32)	14.2 (8.9, 19.4) 0.24 (0.15, 0.33)	18.0 (11.4, 24.6) 0.30 (0.19, 0.41)	< 0.001
Multivariate difference,^c ^μ*M* mg/dl	0 (referent)	7.5 (2.9, 12.2) 0.13 (0.05, 0.21)	13.5 (7.2, 19.9) 0.23 (0.12, 0.34)	14.4 (9.0, 19.8) 0.24 (0.15, 0.33)	18.7 (11.8, 25.7) 0.31 (0.20, 0.43)	< 0.001

^a^Adjusted for age, sex, race, body mass index, use of diuretics, allopurinol and uricosuric agents, and hypertension. ^b^Adjusted for age, sex, race, body mass index, use of diuretics, allopurinol and uricosuric agents, hypertension, serum calcium levels, serum alkaline phosphatase levels, serum phosphorous levels, serum vitamin D levels, and glomerular filtration rate. ^c^Additionally adjusted for total daily intake of alcohol, energy, protein, sugar, and caffeine.

When hyperuricemia was examined as a dichotomous outcome, the relations were similar (Table 3). For example, the multivariate odds ratios (ORs) for hyperuricemia according to increasing quintiles of serum PTH level were 1, 1.07, 1.22, 1.36, and 1.39 (95% CI, 1.03 to 1.88; *P *for trend = 0.03). Alternative definitions of hyperuricemia (serum uric acid levels > 6.0 mg/dl and > 7.0 mg/dl, both regardless of sex) did not materially alter these results (*P *values for trend < 0.03).

**Table 3:** The odds ratios (ORs) of hyperuricemia^a ^according to quintiles of parathyroid hormone in NHANES 2003-2006

Parathyroid hormone levels p*M* (pg/ml)	Quintile 1 0.64-2.86 (6-27)	Quintile 2 2.97-3.82 (28-36)	Quintile 3 3.93-4.67 (37-44)	Quintile 4 4.77-6.15 (45-58)	Quintile 5 6.26-52.10 (59-491)	*P *value for trend
Age- and Sex-adjusted OR	1 (referent)	1.08 (0.77, 1.51)	1.22 (0.84, 1.76)	1.54 (1.14, 2.08)	2.17 (1.62, 2.89)	< 0.001
Multivariate OR^b^	1 (referent)	1.03 (0.75, 1.71)	1.12 (0.77, 1.61)	1.29 (0.97, 1.71)	1.56 (1.165, 2.10)	< 0.001
Multivariate OR^c^	1 (referent)	1.05 (0.76, 1.46)	1.18 (0.81, 1.74)	1.34 (1.02, 1.76)	1.34 (1.01, 1.77)	0.03
Multivariate OR^d^	1 (referent)	1.07 (0.77, 1.49)	1.22 (0.81, 1.82)	1.36 (1.03, 1.80)	1.39 (1.03, 1.88)	0.03

^a ^Hyperuricemia is defined as having a serum uric acid level **> **7.0 mg/dl for men and **> **5.7 mg/dl for women. ^b^Adjusted for age, sex, race, body mass index, use of diuretics, allopurinol and uricosuric agents, and hypertension. ^c^Adjusted for age, sex, race, body mass index, use of diuretics, allopurinol and uricosuric agents, hypertension, serum calcium levels, serum alkaline phosphatase levels, serum phosphorus levels, serum vitamin D levels, and glomerular filtration rate. ^d^Additionally adjusted for total daily intake of alcohol, energy, protein, sugar, and caffeine.

Additionally, when we stratified our multivariate analysis by subgroups of BMI, GFR, serum vitamin D levels, and serum calcium levels, the association between PTH and serum uric acid levels persisted in all subgroups (*P *values for trend ≤ 0.007). The association did not vary significantly by these subgroups (*P *values for interaction > 0.55) except for GFR subgroups. Serum uric acid levels in individuals of the highest quintile of PTH were higher than in the lowest quintile by 26 μ*M *(*P *for trend < 0.001) in the lower GFR group, whereas the corresponding difference was 15 μ*M *(*P *for trend = 0.007) in the higher GFR group (*P *for interaction = 0.004).

## Discussion

In our nationally representative population study of US men and women, we found that serum uric acid levels and the frequency of hyperuricemia increased with increasing PTH levels in a graded manner. These associations were independent of other risk factors for hyperuricemia, such as age, sex, BMI, waist circumference, dietary risk factors, alcohol intake, renal function, hypertension, diuretic use, and serum levels of calcium and vitamin D. The association persisted across subgroups of BMI, serum levels of calcium, and vitamin D, and was greater in the lower GFR group. These findings provide evidence that supports an independent link between serum PTH and uric acid levels at the national population level.

The mean difference in serum uric acid level between the extreme quintiles of PTH was 19 μ*M *(0.31 mg/dl), whereas the difference among individuals with chronic kidney disease (stage 2 or higher) was 26 μ*M *(0.43 mg/dl). These levels of adjusted population mean differences were comparable to difference estimates between the extreme quintiles of intakes of total meats or seafood (0.11 mg/dl versus 0.10 mg/dl) in another NHANES study [19], which can be translated into a significant risk for incident gout, as demonstrated in a prospective analysis of the Health Professionals Follow-up Study [20]. This potential impact on the eventual risk of gout was also supported by our results with various definitions of hyperuricemia as a dichotomous outcome.

The link between PTH, hyperuricemia, and gout was observed in primary hyperparathyroidism [2-7]. For example, in an NIH case series, 20 of 56 patients (not taking diuretics) had serum uric acid levels > 7 mg/dl [3]. After parathyroidectomy, serum uric acid levels decreased by more than 1 mg/dl in 64% of these patients [3]. Limited studies have suggested that reduced renal excretion is responsible for the link, but the detailed underlying process remains unknown [7].

Recently, notable advances were made in the physiology of proximal tubular urate transport, with the identification of multiple secretory and absorptive transporters at both the basolateral and apical membranes [21-24]; urate transport across the proximal tubule is the net sum of these two processes. PTH would be expected to have more-negative effects on net proximal tubular urate reabsorption, given that proximal tubular salt (Na^+^-Cl^-^) and urate transport are regulated in parallel [25] and that PTH is a potent inhibitor of NHE3 [26], a critical apical transporter for proximal tubular salt absorption.

Recent osteoporosis prevention trials of teriparatride as a bone-formation stimulant have also reported hyperuricemia as an adverse event. For example, a clinical trial of 1,637 postmenopausal women found that teriparatride increases the incidence of hyperuricemia in a dose-response fashion, up to 25% at a dose of 40 μg per day [8,9]. After cessation of treatment, serum uric acid levels returned to or approached pretreatment levels [8]. However, a randomized trial of men 40 years or older suggested that reducing PTH levels by calcium supplementation may not affect serum uric acid levels [11]. Interestingly, the hyperuricemia adverse events in the teriparatride trial [8,9] were substantially more frequent among renally impaired patients, which was the case in the current population data. These data suggest that renal impairment or its associated processes significantly influence the hyperuricemic effects of PTH, calling for future investigations to elucidate the underlying mechanisms. Furthermore, this raises an intriguing question of whether vitamin D supplementation in renally impaired patients could help to reduce their serum uric acid levels. Another clinical trial of 428 glucocorticoid-treated adults reported both hyperuricemia (*n *= 3) and gout (*n *= 1) as adverse events of teriparatride, 20 μg per day [10]. Our findings expand these randomized trial data from a specific disease population to a general population level [2-10].

Strengths and limitations of our study deserve comment. This study was performed in a nationally representative sample of US men and women; thus the findings are likely to be generalizable to the US adult population. Although our cross-sectional study alone would leave uncertainty regarding the temporal sequence of exposure-outcome relations and thus about a potential causal link, the aforementioned randomized trial data with dose-response relation and reduction of uric acid levels after cessation of exposure (that is, teriparatride) or after parathyroidectomy in primary hyperparathyroidism, collectively provide a strong causal argument. Thus, our study findings should be viewed as expanding the generalizability of these previous findings to the general population.

Regardless, confirming the relation in a prospective longitudinal observational context (for example, the relation between prior PTH levels and incident gout, particularly in a renally impaired group) would be valuable.

## Conclusions

These nationally representative data indicate that serum PTH levels are independently associated with serum uric acid levels and the frequency of hyperuricemia at the general population level. These data provide support for future studies aimed to clarify the underlying molecular mechanisms.

## Abbreviations

BMI: body mass index; GFR: glomerular filtration rate; NHANES: National Health and Nutritional Examination Survey; PTH: parathyroid hormone; SUA: serum uric acid.

## Competing interests

Dr. Choi served on advisory boards for Takeda Pharmaceuticals, URL Pharma, and Savient Pharmaceuticals, whereas the other authors have nothing to disclose.

## Authors' contributions

All authors contributed to studying the concept and design, acquisition of data, analysis and interpretation of data, and critical revision of the manuscript for important intellectual content. JH, JWC, and HC drafted the manuscript, and all authors read and approved the final manuscript.
